# Rare manifestations of Potter Sequence: A Case Report

**DOI:** 10.31729/jnma.4683

**Published:** 2020-03-31

**Authors:** Uttara Gautam, Rishikesh Kafley, Vijay Chikanbanjar, Alyssa Shakya, Rydam Basnet, Sunil Raja Manandhar

**Affiliations:** 1 Neonatal Unit, Department of Pediatrics, Kathmandu Medical College Teaching Hospital, Sinamangal, Kathmandu, Nepal

**Keywords:** *oligohydramnios*, *Potter Sequence*, *Potter's facies*, *pulmonary hypoplasia*

## Abstract

Potter sequence is a rare congenital malformation that primarily affects male fetuses and is characterized by pulmonary hypoplasia, skeletal malformation, and kidney abnormalities. The pressure of the uterine wall due to oligohydramnios leads to an unusual facial appearance, abnormal limbsor limbs in abnormal positions or contractures. The fetus generally dies soon after birth due to respiratory insufficiency. We presented a male baby of 35 wks gestation with birth weight 1200gms delivered by primi mother. She had severe oligohydramnios and virtually there was no liquor during birth. The baby had severe perinatal depression at birth requiring resuscitation. Multiple congenital anomalies like absence of left eye, congenital cataract on the right eye, right-sided choanal atresia, micrognathia, low set ears, beaked nose, bilateral clubbed foot with hip deformity were noted. After 2 hours of life,baby developed fast breathing and cyanosis and died due to respiratory failure.

## INTRODUCTION

Potter sequence was first described by Edith Potter 1946 at the Chicago Lying Hospital in the U.S.A that consisted of facial characteristics in infants with bilateral renal agenesis.^1^ The sequence with an incidence of 1 in every 2,000 to 5,000 fetuses is associated with a recurrence risk of 3-6% and is found in 0.2-0.4% of autopsies in dead new-borns or those who die immediately after birth.^2^ It includes clubbed feet, pulmonary hypoplasia and cranial anomalies related to oligohydramnios. The Potter sequence is due to the restricted ability for certain organs to grow due to severe oligohydramnios.

Oligohydramnios is very common and is the cause of deformities observed in Potter sequence leading to decreased urine production secondary to bilateral renal agenesis, obstruction of the urinary tract or occasionally, prolonged rupture of membranes.^3^ The resulting oligohydramnios is the cause of the typical facial appearance of the fetus, which is known as “Potter's facies” which consists of a flattened nose, recessed chin, epicanthal folds and low-set abnormal ears.^4^

The main cause of the Potter sequence is unknown and this sequence has a genetic background in some cases and is more common in neonates with a family history of kidney abnormalities.^5^

## CASE REPORT

A 22 years old primi mother delivered a preterm, male baby of 35 wks gestation with birth weight 1200gms via spontaneous vaginal delivery. There was no liquor and both feto-maternal surfaces of the placenta were normal. Baby didn't cry immediately at birth and the baby was flaccid with flexed hip posture. After tactile stimulation, Bag and Mask ventilation were started as babydid not have respiratory effort. APGAR score of the baby was 5/10 and 6/10 at 1 and 5 minutes respectively. Multiple congenital anomalies were noted. The facial features noted in this baby were low set ears with overturned helix, absence of whole right orbital cavity along with right eyeball, congenital cataract of the left eye, Mongolian slanting of the left eye, flatten broad ear, flatter nasal bridge, beaked nose, rightsided choanal atresia and micrognathia. There was the persistence of flexed hip posture due to hip contracture and dislocation with bilateral club feet. After 15mins of life, the baby developed tachypnea and severe chest retraction not maintaining saturation with oxygen. Supportive conservative treatment was done. After two hours of birth, the baby died due to respiratory insufficiency.

**Figure 1:**
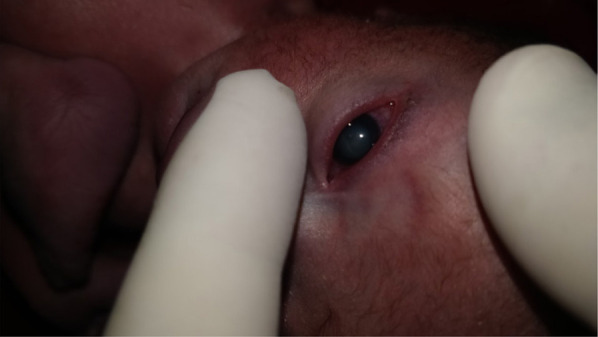
Showing congenital cataract.

**Figure 2:**
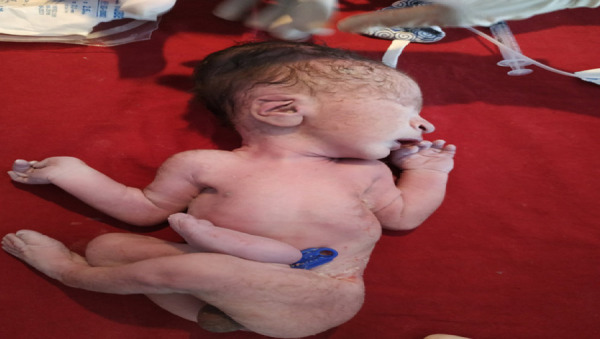
Showing low set ear, absence of right eye ball including orbital cavity , beaked nose and micrognathia.

**Figure 3:**
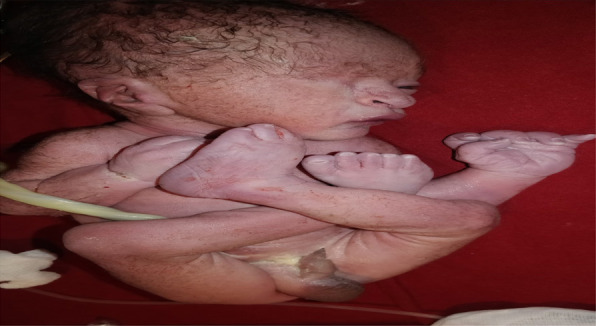
Showing persistent flexed and dislocated hip with bilateral clubbed foot.

The primi mother had ANC checkup at a primary health center of Dolakha district, Nepal. Family history was insignificant for any medical and surgical illness including renal diseases.In-utero ultra-sonogram (USG) scan at 18 wks gestation showed anhydramnios, but pregnancy was continued. At 35 wks gestation the mother was brought to Kathmandu Medical College Teaching Hospital with labor pain where she delivered a live baby vaginally. The diagnosis of Potter sequence was done based onvisible physical findings of the baby. Post-death USG abdomen and postmortemof this baby could not be done due to the parental ritual aspect.

## DISCUSSION

This case report presents a Potter sequence with rarer manifestations such as absence of one eye including orbit, unilateral choanal atresia. This probably has not been reported in Nepal so far. It was a fatal case of Potter sequence born through vaginal delivery. Despite the amniotic fluid index being zero from the 18th week of gestation, pregnancy was continued till 35 weeks and a male baby weighing 1.2kg was born with typical Potter's facies that died shortly after birth.

Potter sequence describes the typical physical appearance caused by pressure in utero due to oligohydramnios. It can cause other abnormalities like infantilepolycystic kidney disease, renal hypoplasia, and obstructive uropathy. Potter sequence can also have congenital abnormalitiesdue to the prolonged leakage of amniotic fluid during the middle gestational weeksand may have normal kidneys also.^6^ The severity of the pulmonary hypoplasia depends on the developmental phase of the lung in which oligohydramnios occurs due to its intensity and duration of the oligohydramnios. Because of severe respiratory distress and lung hypoplasia, fetuses are born with dysplastic kidneys or die shortly after birth.^7^

Babies of Potter sequence do not have the same set of signs, but they share a common chain of events leading to the same endpoint of reduced or absent amniotic fluid. The decrease in the volume of amniotic fluid may be due to decreased urine production secondary to bilateral renal agenesis, obstruction to the urinary tract or occasionally prolonged rupture of membranes. The resulting oligohydramnios is the cause of the deformities in Potter sequence.^7^

The main cause of the Potter sequence remains unclear in most cases, but it has a genetic reason in some cases, and the inheritance pattern depends on a particular genetic cause. Genetic abnormalities such as autosomal dominant or recessive inheritance of polycystic kidney disease, hereditary kidney dysplasia, caused by RET and UPK3A gene mutations and chromosomal abnormalities, can result in developmental abnormalities and lead to Potter sequence. This sequence occurs sporadically but may be inherited when arising from the autosomal dominant triad. Potter sequence is more common in babies who have a family history of kidney abnormalities.^4,8^

The evaluation of baby's with Potter's sequence should include an examination for non-renal defects, autopsy, chromosome analysis and renal ultrasound or urologic evaluation. Ultrasonographic prenatal monitoring of subsequent pregnancies in such families is strongly recommended because of a definite but unknown degree of recurrence risk. Regular follow-up and antenatal checkups should be done to diagnose oligohydramnios to prevent the complications resulting from it. Not all oligohydramnios cases will lead to Potter's sequence, but detail anomaly scan of the fetus and regular amniotic fluid index should be done to identify these types of sequences.^9^

Potter sequence refers to the typical facial characteristics and associated pulmonary hypoplasia of a neonate as a direct result of oligohydramnios due to the renal pathology. It usually affects the male baby and severe respiratory insufficiency leads to a fatal outcome in most of the babies. Prenatal USG helps to detect this sequence early by examining oligohydramnios and kidney conditions.
